# Which is the top player for the cardiovascular safety? ibrutinib vs. obinutuzumab in CLL

**DOI:** 10.3389/fphar.2023.1229304

**Published:** 2023-08-16

**Authors:** Annamaria Mascolo, Raffaella Di Napoli, Nunzia Balzano, Elena D’Alessio, Imma Izzo, Francesco Rossi, Giuseppe Paolisso, Annalisa Capuano, Liberata Sportiello

**Affiliations:** ^1^ Campania Regional Centre for Pharmacovigilance and Pharmacoepidemiology, Napoli, Italy; ^2^ Department of Experimental Medicine—Section of Pharmacology “L. Donatelli”, University of Campania “Luigi Vanvitelli”, Napoli, Italy; ^3^ Pharmacy Unit, Ospedale del Mare—A.S.L. Na1-Centro, Naples, Italy; ^4^ Department of Advanced Medical and Surgical Sciences, University of Campania “Luigi Vanvitelli”, Naples, Italy

**Keywords:** ibrutinib, obinutuzumab, chronic lymphocytic leukemia, cardiovascular safety, pharmacovigilance, real-world data

## Abstract

**Introduction:** Ibrutinib, a Bruton’s tyrosine kinase (BTK) inhibitor, is authorized for the treatment of chronic lymphocytic leukemia (CLL). This study aims to explore the cardiac safety profile of ibrutinib in comparison with obinutuzumab.

**Methods:** A retrospective pharmacovigilance study was conducted on data retrieved from the European pharmacovigilance database (Eudravigilance) from 1 January 2014 to 30 September 2022. To compare the reporting frequency of cardiovascular events among ibrutinib, obinutuzumab, and the combination of both.

**Results:** A total of 2 291 CV cases were retrieved, of which 1965 were related to ibrutinib, 312 to obinutuzumab, and 14 to the combination. Most cases referred to patients aged ≥65 years (*N* = 1,454; 63.47%) and male (*N* = 1,497; 65.34%). Most cases were serious (*N* = 2,131; 93.02%). The most reported events were: atrial fibrillation (*N* = 913; 31.31%) and haemorrhage (*N* = 201; 6.89%). A higher reporting frequency of CV events was found when ibrutinib was compared to obinutuzumab (ROR, 3.22; 95% CI, 2.89-3.60) or combination (ROR, 1.77; 95% CI, 1.11-2.83). A lower reporting was observed when obinutuzumab was compared to combination (ROR, 0.55; 95% CI, 0.34-0.88).

**Discussion:** A higher reporting frequency of CV events in patients exposed to ibrutinib in comparison with obinutuzumab was found. Further studies are needed to better explore the safety of ibrutinib.

## 1 Introduction

Chronic lymphocytic leukemia (CLL) is the most common type of blood cancer in adults characterized by specific genomic alterations that affect B-cells proliferation and apoptosis (Maddocks and Jones, 2016; Burger et al., 2019). Frontline therapies for CLL include chemoimmunotherapy regimens, monoclonal CD20 antibodies, inhibitors of the Bruton’s tyrosine kinase (BTK), and inhibitor of the B-cell lymphoma 2 (BCL2) protein (Eichhorst et al., 2021). Among them, BTK inhibitors represent a promising therapeutic strategy demonstrating a positive clinical impact in patients with CLL. Indeed, BTK is a kinase downstream of the B-cell receptor (BCR) signalling pathway that, when inhibited, negatively influences the B-cell differentiation, migration, and proliferation (Maddocks and Jones, 2016). Ibrutinib, a potent oral first-in-class BTK inhibitor, is authorized in Europe since 2014 for the treatment of adult patients with previously untreated CLL or who received at least one prior therapy for CLL (European Medicines Agency (EMA), 2019). Despite its efficacy, it has been associated with the onset of clinically significant adverse events, such as diarrhoea, arthralgia, haemorrhage, infection, cardiac arrhythmia, and hypertension (Broccoli et al., 2021). Among them, cardiovascular (CV) events have become of particular concern over the years. Clinical trials highlighted the occurrence of cardiac events, such as hypertension and atrial fibrillation, following the administration of ibrutinib in adult patients with CLL (Caldeira et al., 2019). A real-world study also showed a higher reporting of cardiac conduction abnormalities, haemorrhagic events, heart failure, and hypertension (Salem et al., 2019). Moreover, ventricular arrhythmias and sudden deaths have been associated with the administration of ibrutinib, with an estimated incidence rate of sudden death of 788 events per 100,000 person-years (Lampson et al., 2017). Generally, toxicity rates of ibrutinib did not significantly differ between drug doses (Uminski et al., 2019). An analysis of real-world data from the World Health Organization pharmacovigilance database revealed potentially relevant risk factors for ibrutinib-associated cardiac events, including the advanced age (<75 years and >75 years) and the history of cardiac co-morbidities (Allouchery et al., 2021). Considering the clinical significance of these events, the European Medicines Agency (EMA) published a direct healthcare professional communication (DHPC) with the aim of minimizing the cardiac risk and informing about the main factors contributing to ibrutinib-related cardiotoxicity (European Medicines Agency EMA, 2022). Indeed, alternative drugs may be considered in patients with risk factors for cardiac events. However, the evidence comparing the safety of ibrutinib with other CLL effective treatments in real-life settings is meagre, especially on long-term clinical evaluation. Therefore, we decided to conduct a European pharmacovigilance study to further explore the cardiac safety profile of ibrutinib in comparison with another recommended drug for CLL, the anti-CD20 obinutuzumab.

## 2 Materials and methods

### 2.1 Study design

A retrospective pharmacovigilance study comparing the CV safety profile of ibrutinib with obinutuzumab in CLL.

### 2.2 Data source

The European pharmacovigilance database (Eudravigilance, EV), available at www.adrreports.eu, was used to retrieve Individual Case Safety Reports (ICSRs) from 1 January 2014 to 30 September 2022. The EV is a database managed by the EMA to organize and analyse ICSRs related to medicines or vaccines authorized or under investigation in clinical trials in the European Economic Area (EEA). Healthcare professionals (HCP) or non-HCP can spontaneously report an ICSR to an EU national competent authority or a marketing authorization holder responsible for their entering in EV.

### 2.3 ICSRs selection

ICSRs were selected by using the line listing function of EV for the suspected drugs ibrutinib or obinutuzumab. A suspected drug is a drug taken by the patient and suspected by the reporter to have determined the adverse reactions. It is different from a concomitant drug, which is any medicine taken by the patient at the time the adverse reaction is observed, but not responsible for the reaction itself. ICSRs selection was restricted to only cases with ibrutinib or obinutuzumab used for CLL. CV ICSRs were identified based on the presence of at least one CV event. CV events were identified by using the System Organ Classes (SOCs) of the Medical Dictionary for Regulatory Activities (MedDRA): “Cardiac disorders” and “Vascular disorders.” ICSRs were grouped based on the suspected drug into: “cases with ibrutinib,” “cases with obinutuzumab,” or “cases with both ibrutinib and obinutuzumab (here on indicated as combination).”

### 2.4 Descriptive analysis

ICSRs were analyzed for patient’s characteristics (age group and sex), seriousness of the case, type of reporter (HCP or non-HCP), primary Source Country for regulatory purposes (EEA or non-EEA), number of reported suspected drugs (classified as 1, 2, 3 or ≥4) and concomitant drugs (classified as 0, 1, 2, 3 or ≥4). The 1, 2, 3, or ≥4 were the numbers of suspected or concomitant drugs reported in each ICSRs. The 0 was attributed when no concomitant drug was reported. CV ICSRs were also described for the presence of “sudden death” among reported Preferred Term (PT). All CV events were tabled for ibrutinib, obinutuzumab, and combination and described in terms of PT, outcome and seriousness. According to MedDRA, each PT was a specific medical concept indicative of a symptom, sign, or diagnosis, and it was subordinate to a higher level, defined as “High-Level Group Terms” (HLGTs). The seriousness was classified in accordance with the International Council on Harmonization E2D guidelines. An event was serious if resulting in death, hospitalization or its prolongation, severe or permanent disability, or congenital abnormalities/birth deficits, as well as if it was life-threatening or a clinically relevant condition. The outcome was classified as “recovered/resolved,” “recovering/resolving,” “recovered/resolved with sequelae,” “not recovered/not resolved,” “fatal,” and “unknown.” The annual reporting trend of CV ICSRs and the annual percentage change were also computed to see possible differences during COVID-19 pandemic. The annual trends and the annual percentage changes were estimated overall, for ibrutinib, and obinutuzumab. Spontaneous reporting trends were also calculated for fatal outcomes.

### 2.5 Disproportionality analyses

To compare the reporting frequency of the overall CV events among ibrutinib, obinutuzumab, and the combination of both, the Reporting Odds Ratio (ROR), its 95% Confidence interval (95%CI), and the chi-square test were computed. Moreover, the RORs and their 95%CI were also calculated for the four overall most reported CV events. Finally, the RORs and their 95%CI were computed to evaluate the reporting frequency of overall CV events and four most reported events between age groups (≥65 years vs. < 65 years) among ibrutinib ICSRs. Disproportionality analyses were performed only if at least 3 events were reported for each treatment. A 5% significance level was considered for all analyses. RORs were displayed with forest plots performed by using R (version 3.2.2, R Development Core Team).

## 3 Results

### 3.1 Descriptive results

**FIGURE 1:**
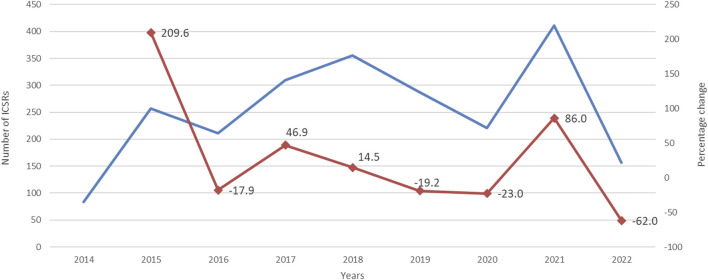
Trends of cardiovascular spontaneous reporting from EudraVigilnce database between 1 January 2014 and 30 September 2022.

During study period, a total of 12,631 ICSRs reporting ibrutinib or obinutuzumab (8,302 for ibrutinib, 4,252 for obinutuzumab, and 77 for combination) as suspect drug in CLL patients were sent to the Eudravigilance database (data not shown). Among these ICSRs, a total of 2,291 CV ICSRs were retrieved, of which 1,965 were related to ibrutinib, 312 to obinutuzumab, and 14 to the combination (ibrutinib/obinutuzumab). The annual spontaneous reporting trend showed the highest peak in 2015, followed by a constant pattern between 2016-2020. However, there was an increase of spontaneous reporting in 2021 compared to 2020 (+86.0%; Figure 1). This trend was mainly driven by ibrutinib reporting (Supplementary Figure S1), while for obinutuzumab a decreased reporting was observed during years 2020-2021 (Supplementary Figure S2). Most ICSRs referred to patients aged ≥65 years (*N* = 1,454; 63.47%) and male (*N* = 1,497; 65.34%). Most ICSRs were serious (*N* = 2,131; 93.02%) and reported by HCPs (*N* = 1,930; 84.24%). The 66% of ICSRs reported only ibrutinib or obinutuzumab as suspected drug and the 56.70% did not report any concomitant medication. Venetoclax and/or chlorambucil were reported as suspected drugs in 146 ICSRs (6.4%), and as concomitant drugs in 54 ICSRs (2.4%). Specifically, for suspected drugs, 22 ICSRs related to ibrutinib and 60 related to obinutuzumab reported chlorambucil; 52 ICSRs related to ibrutinib and 11 related to obinutuzumab reported venetoclax; 1 ICSRs related to both ibrutinib and obinutuzumab reported venetoclax. For concomitants, 43 ICSRs related to obinutuzumab and 3 ICSRs related to ibrutinib reported chlorambucil; 6 ICSRs related to ibrutinib and 1 related to obinutuzumab reported venetoclax; 1 ICSRs related to ibrutinib reported both venetoclax and chlorambucil. Characteristics of CV ICSRs for ibrutinib, obinutuzumab or the combination are reported in Table 1. Among ICSRs, a total of 2,916 CV events were identified with a mean number of 1.27 events per ICSRs. Only one case reported sudden death associated with ibrutinib administration, and no case was found for obinutuzumab. Analyzing data by MedDRA HLGTs, the majority of CV events were categorized as “Cardiac arrhythmias” (*N* = 1,344; 46.09%), “Vascular haemorrhagic disorders” (*N* = 326; 11.18%), “Decreased and nonspecific blood pressure disorders and shock” (*N* = 193; 6.62%), and “Vascular hypertensive disorders” (*N* = 192; 6.58%). Most events occurred with ibrutinib alone (*N* = 2,514; 86.21%), followed by obinutuzumab alone (*N* = 383; 13.13%) and combination (*N* = 19; 0.65%). Overall, the four most reported events were: atrial fibrillation (*N* = 913; 31.31%), haemorrhage (*N* = 201; 6.89%), hypertension (*N* = 173; 5.93%), and hypotension (*N* = 160; 5.49%). CV events are listed in Table 2 and Supplementary Table S1. Moreover, hypotension was reported in 160 ICSRs as “hypotension” and in 8 ICSRs as “orthostatic hypotension”. Among these cases, we seek for concomitant medications. We found 470 concomitant drugs, of which 117 belongs to the Anatomical-Therapeutic-Chemical (ATC) code “Cardiovascular system” (ATC code: C). Diphenhydramine was concomitantly reported in only two cases (1 for ibrutinib and 1 for obinutuzumab). A total of 2,691 (92.28%) CV events met seriousness criteria. The criterion most reported was other medically important condition (41.12%), followed by caused/prolonged hospitalization (33.88%), results in death (10.91%), life threatening (5.80%), and disabling (0.57%). The outcome was reported for 1,681 (57.65%) CV events and was positive (recovered/resolved or recovering/resolving) for 993 events (34.05%). However, 10.91% of CV reactions had a fatal outcome. Analyzing the annual reporting trend of fatal outcomes, there was a highest increase in 2021 (+346.7%, data shown in Supplementary Figure S3). Nevertheless, only 6 ICSRs reported also COVID-19 infection as ADR of which 3 cases had a fatal outcome. Specifics of seriousness and outcome criteria are reported in Table 3.

**TABLE 1:** Distribution for age group, sex, seriousness, primary source, primary source country for regulatory purposes, presence of other suspected or concomitant drugs among Individual Case Safety Reports (ICSRs) related to cardiac and vascular events in patients treated with ibrutinib and/or obinutuzumab reported in Eudravigilance from January 1st. 2014 to September 30st. 2022.

Characteristics	Level	Ibrutinib (*n* = 1965; 85.77%)	Obinutuzumab (*n* = 312; 13.62%)	Ibrutinib/Obinutuzumab (*n* = 14; 0.61%)	Total (*n* = 2291; 100%)
Number of events per ICSR	Mean	4.01	4.13	8.71	4.06
Age Group	18-64 years	325 (16.54)	34 (10.90)	7 (50.00)	366 (15.98)
	65-85 Years	1097 (55.83)	185 (59.29)	5 (35.71)	1287 (56.18)
	More than 85 Years	156 (7.94)	11 (3.53)	—	167 (7.29)
	Not specified	387 (19.69)	82 (26.28)	2 (14.29)	471 (20.56)
Sex	Female	621 (31.60)	102 (32.69)	5 (35.71)	728 (31.78)
	Male	1297 (66.01)	191 (61.22)	9 (64.29)	1497 (65.34)
	Not Specified	47 (2.39)	19 (6.09)	—	66 (2.88)
Seriousness of ICSR	Serious	1834 (93.33)	283 (90.71)	14 (100.00)	2131 (93.02)
	Not serious	131 (6.67)	29 (9.29)	—	160 (6.98)
Primary Source	Healthcare Professional	1618 (82.34)	300 (96.15)	12 (85.71)	1930 (84.24)
	Non-Healthcare Professional	347 (17.66)	12 (3.85)	2 (14.29)	361 (15.76)
Primary Source Country for Regulatory Purposes	European Economic Area	835 (42.49)	154 (49.36)	1 (7.1-4)	990 (43.21)
	Non-European Economic Area	1130 (57.51)	158 (50.64)	13 (92.86)	1301 (56.79)
Suspected drug(s)	1	1312 (66.77)	200 (64.10)	—	1512 (66.00)
	2	386 (19.64)	80 (25.64)	11 (78.57)	477 (20.82)
	3	145 (7.38)	10 (3.21)	—	155 (6.77)
	≥4	122 (6.21)	22 (7.05)	3 (21.43)	147 (6.42)
Concomitant drug(s)	0	1103 (56.13)	186 (59.62)	10 (71.43)	1299 (56.70)
	1	117 (5.95)	31 (9.94)	1 (7.14)	149 (6.50)
	2	119 (6.06)	13 (4.17)	—	132 (5.76)
	3	101 (5.14)	17 (5.45)	—	118 (5.15)
	≥ 4	525 (26.72)	65 (20.83)	3 (21.43)	593 (25.88)

**TABLE 2:** Cardiovascular events categorized by HLGTs listed in ICSRs with ibrutinib and/or obinituzumab as suspected drug and reported in Eudravigilance from January 1st. 2014 to September 30st. 2022.

HLGT	Ibrutinib	Ibrutinib/Obinituzumab	Obinituzumab	Total
Aneurysms and artery dissections	12	0	0	12
Arteriosclerosis, stenosis, vascular insufficiency and necrosis	33	0	6	39
Cardiac arrhythmias	1241	8	95	1344
Cardiac disorders, signs and symptoms NEC	94	2	18	114
Cardiac valve disorders	17	0	3	20
Coronary artery disorders	102	1	43	146
Decreased and nonspecific blood pressure disorders and shock	73	2	118	193
Embolism and thrombosis	57	2	2	61
Heart failures	186	1	14	201
Lymphatic vessel disorders	14	0	1	15
Myocardial disorders	65	1	2	68
Pericardial disorders	97	0	1	98
Vascular disorders NEC	29	0	41	70
Vascular haemorrhagic disorders	321	0	5	326
Vascular hypertensive disorders	157	2	33	192
Vascular infections and inflammations	14	0	1	15
Venous varices	2	0	0	2

**TABLE 3:** Distribution for seriousness and outcomes of cardiovascular events reported in ICSRs with ibrutinib and/or obinutuzumab as suspected drug.

	Number of events with ibrutinib (*n* = 2514; 86.22%)	Number of events with obinutuzumab (*n* = 383; 13.13%)	Number of events with ibrutinib/obinutuzumab (*n* = 19; 0.65%)	Number of cardiovascular events (*n* = 2916; 100%)
Seriousness				
*Not available*	176 (7.00)	49 (12.79)	—	225 (7.72)
*Serious*	2338 (93.00)	334 (87.21)	19 (100%)	2691 (92.28)
Seriousness Criteria				
*Other Medically Important Condition*	1061 (42.20)	133 (34.73)	5 (26.32)	1199 (41.12)
*Results in Death*	256 (10.18)	58 (15.14)	4 (21.05)	318 (10.91)
*Caused/Prolonged Hospitalisation*	862 (34.28)	116 (30.29)	10 (52.63)	988 (33.88)
*Life Threatening*	142 (5.65)	27 (7.05)	—	169 (5.80)
*Disabling*	17 (0.68)	—	—	17 (0.57)
Outcome				
*Recovered/Resolved*	569 (22.63)	158 (41.25)	4 (21.05)	731 (25.07)
*Recovering/Resolving*	246 (9.79)	16 (4.18)	—	262 (8.98)
*Recovered/Resolved with Sequelae*	22 (0.88)	3 (0.79)	—	25 (0.86)
*Not Recovered/Not Resolved*	327 (13.00)	16 (4.18)	2 (10.53)	345 (11.83)
*Fatal*	256 (10.18)	58 (15.14)	4 (21.05)	318 (10.91)
*Unknown*	1094 (43.52)	132 (34.46)	9 (47.37)	1235 (42.35)

### 3.2 Results from disproportionality analyses

**FIGURE 2:**
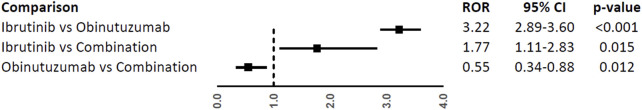
Reporting Odds Ratios (RORs) of cardiovascular events comparing ibrutinib vs. obinituzumab, and ibrutinib or obinituzumab vs. combination of both.

A higher reporting frequency of CV events was found when ibrutinib was compared to obinutuzumab (ROR, 3.22; 95%CI, 2.89-3.60) or combination (ROR, 1.77; 95%CI, 1.11-2.83), while a lower reporting was observed when obinutuzumab was compared to combination (ROR, 0.55; 95%CI, 0.34-0.88) (Figure 2).

**FIGURE 3:**
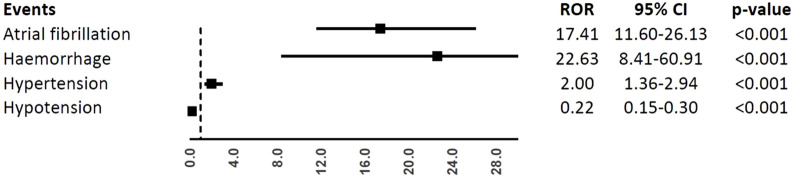
Reporting Odds Ratios (RORs) of atrial fibrillation, haemorrhage, hypertension, hypotension comparing ibrutinib vs. obinituzumab.

In the analyses of specific CV events, ibrutinib was associated with a higher reporting frequency of atrial fibrillation (ROR, 17.41; 95%CI, 11.60-26.13), haemorrhage (ROR, 22.63; 95%CI, 8.41-60.91), and hypertension (ROR, 2.00; 95%CI, 1.36-2.94), but not hypotension (ROR, 0.22; 95%CI, 0.15-0.30), when compared to obinutuzumab (Figure 3).

**FIGURE 4:**
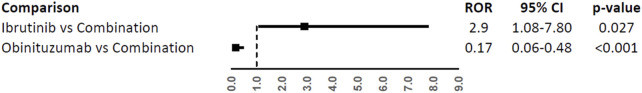
Reporting Odds Ratios (RORs) of atrial fibrillation comparing ibrutinib or obinituzumab vs. combination of both.

In the comparison with combination (Figure 4), only atrial fibrillation was reported at least 3 times with a higher reporting frequency with ibrutinib (ROR, 2.90; 95%CI, 1.08-7.80) and a lower reporting frequency with obinutuzumab (ROR, 0.17; 95%CI, 0.06-0.48).

**FIGURE 5:**
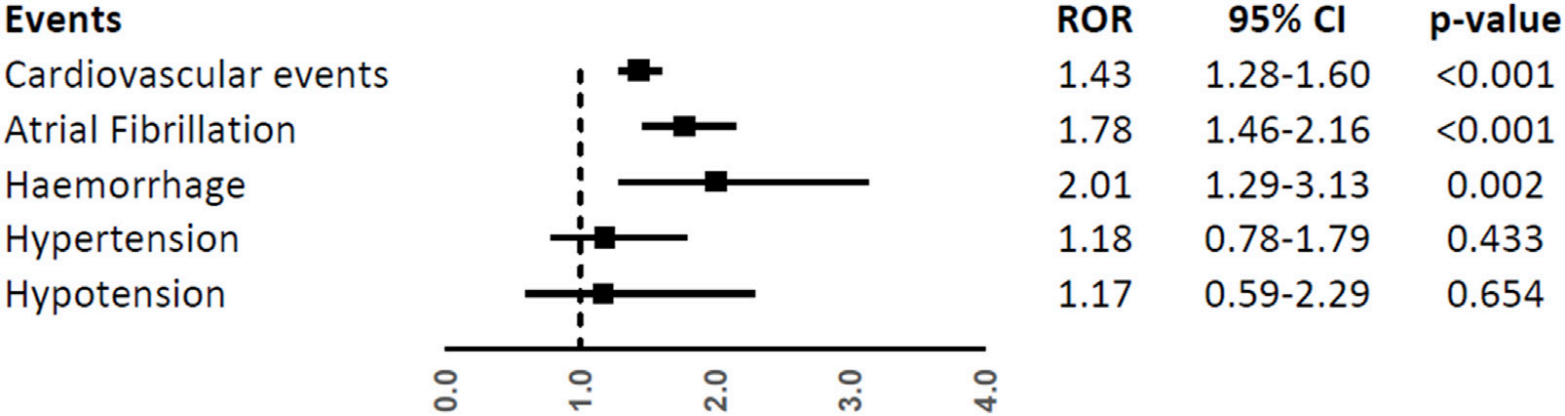
Reporting Odds Ratios (RORs) of cardiovascular events, atrial fibrillation, haemorrhage, hypertension, and hypotension comparing the age groups ≥65 years vs. < 65 years among ibrutinib ICSRs.

Patients aged ≥65 years had a higher reporting frequency of the overall CV events (ROR, 1.43; 95%CI, 1.28-1.60), atrial fibrillation (ROR, 1.78; 95%CI, 1.46-2.16), and haemorrhage (ROR, 2.01; 95%CI, 1.29-3.13), while no difference was found for hypertension and hypotension (ROR, 1.18; 95%CI, 0.78-1.79 and ROR, 1.17; 95%CI, 0.59-2.29) (Figure 5).

## 4 Discussion

The purpose of this study was to compare the CV toxicity of ibrutinib, the first BTK inhibitor, with obinutuzumab, an anti-CD20 of next-generation, in patients affected by CLL. To this aim, we analysed the data reported in the European spontaneous reporting database during almost a decade (from 2014 to 2022). This database gave us the opportunity to assess the CV profile of these two drugs in European Countries, where CLL-related incidence cases increased significantly from 134.28 (123.30–142.03) in 1990 to 275.60 (235.01–338.18) in 2019, with age-standardized incidence rate (ASIR) rising from 3.49/100,000 (3.21–3.69) individuals in 1990 to 6.32/100,000 (5.39–7.75) individuals in 2019 (Western Europe) (Yao et al., 2022).

Although the spontaneous reporting systems are often suffering from incomplete information, we considered all 2,291 reports retrieved from EV because they contained minimum relevant information for the evaluation process.

The annual spontaneous reporting trend showed an increase of spontaneous reporting in 2021 compared to 2020, probably mainly referred to COVID-19 pandemic. This trend mainly driven by ibrutinib suggested a possible role for COVID-19 in precipitating ibrutinib-associated cardiac toxicity. On the contrary, for obinutuzumab a decreased reporting was observed during years 2020-2021, probably due to the lower probability of causing cardiac events and to the peculiar route of administration (intravenous) that requires a specialized environment under the supervision of a Medical Doctor, which was of difficult access during the pandemic.

It is important to underline that the role of the reporters in communicating adverse drug reactions (ADRs) is unavoidably different according with their competency or reporter category (Richard and Roy, 1986; Hunt and Gjoka, 2003; van Grootheest and de Jong-van den Berg, 2004; Blenkinsopp et al., 2007; Parretta et al., 2014). In fact, HCPs can directly or indirectly report a diagnosis or detailed clinical symptoms and signs, whereas non-HCPs such as patients are obviously only able to describe the events. Anyhow, apart from the different professional competence, the active participation to a pharmacovigilance system implies the awareness by any HCP about the occurrence of adverse drug events and the acquirement of a reporting culture (Rafaniello et al., 2016; Scavone et al., 2016; Sessa et al., 2016; 2017). More than 80% of the ICSRs related to ibrutinib and/or obinutuzumab were sent by HCPs. This finding is in line with the closely supervision by specialists in oncology and the particular route of administration for obinutuzumab (intravenous infusion) that requires particular settings where resuscitation equipment is readily available. However, given the oncological conditions of these patients, the 15.76% of their contribution in the reporting is relevant and, according with EMA regulations, it underlines the role of patients as key elements of a pharmacovigilance system and the importance of improving their involvement (European Commission, 2010; Eurpean Commission, 2010).

Most ICSRs (63.47%) involved patients with age more than 65 years and this is in line with the median age of 72 years at the CLL diagnosis (Eichhorst et al., 2021), and the increase of cardiovascular risk with ageing (Kjeldsen, 2018).

Regarding the distribution of ICSRs by sex, we found a higher percentage (more than 60% for both drugs) of male patients experiencing CV events related to these drugs, which seems to be in contrast with the literature data reporting a higher risk of ADRs for women, mainly due to sex-related factors in the pharmacokinetics and pharmacodynamics of drugs (Rademaker, 2001; Zopf et al., 2008). However, more male than female patients (1.9:1) are affected by CLL, and this sex effect seems to be stable across all ethnicities (National Cancer Institute, 2022).

Other suspected and concomitant drugs were reported in almost 35% and 44% of ICSRs, respectively. These data are not surprising considering that oncological patients usually require multiple cancer treatments (Schleicher et al., 2018; Elbeddini et al., 2021). Venetoclax and chlorambucil are widely used in association with obinutuzumab for CLL. The regimen chlorambucil and obinutuzumab is well established and showed efficacy in CLL patients, especially in those who are elderly or with comorbidities. More recently, venetoclax plus obinutuzumab was found superior in terms of progression free survival to chlorambucil plus obinutuzumab. Moreover, these fixed duration therapies are safer than continuous ibrutinib monotherapy (Fischer et al., 2019). However, we found these drugs reported as suspected or concomitant in only 200 ICSRs, corresponding to 8.7% of CV cases.

Therefore, the role of the other medications or comorbidities in the occurrence of CV events could not be excluded. Anyway, no causality assessment between drug administration and the onset of CV event has been performed. Thus, our data are limited by the absence of a final case-by-case evaluation for causality. The lack of information on comorbidities also limited our data and analyses. Patients with CLL often are affected by other diseases, including CV comorbidities (such as heart failure, hypertension, cardiomyopathy, arrhythmia, etc.), that can influence the type of drug to be administered. Moreover, this limitation affects even more any conclusion on CV comorbidities, if we consider that clinical trials typically do not enrol frail patients with significant CV risks. Thus, limiting the comprehension of the relationship between CV comorbidities and ibrutinib-related CV events in clinical practice (Mato et al., 2023). Therefore, real-world studies are needed to also consider patients’ CV comorbidities in the safety analyses of different therapies. Nowadays, current guidelines suggest to prefer obinutuzumab in unfit patients with CV comorbidities (Eichhorst et al., 2021).

More than 90% of CV events related to ibrutinib, obinutuzumab, or their combination were reported as serious and, when the information was filled in, they resulted in complete resolution and improvement. However, our findings also highlighted that in more than 10% of cases there was a fatal outcome, with a peak of reporting in the 2021. This is in line with the major number of spontaneous reporting recording in this year and could be linked with the COVID-19 pandemic period. Obviously, the underlying severe medical condition of these patients could also explain this outcome. However, the real severity of CLL and its clinicopathological characteristics were not available from ICSRs, thus limiting a complete evaluation of our safety data. Indeed, clinicopathological features of CLL, such as levels of β2-microglobulin, ZAP-70 and CD38 expressions, mutation status of the immunoglobulin heavy chain variable region (IGHV), and other genetic mutations, are recognized to influence the disease prognosis and could be linked to the fatal outcome (Shuai et al., 2020). In literature, fatal events related to ibrutinib were reported (Salem et al., 2019). Besides the association with drug exposure, we did not know additional risk factors due to limited information reported in our ICSRs.

More ICSRs related to ibrutinib (*n* = 8,302) than obinutuzumab were reported in the EV database (*n* = 4,252), apart from their combination. This finding could depend on the different usage of these drugs. Despite the lack of data on their use in Europe; we can look from Italian data as a reference. In fact, the last National Report on Medicines use in Italy (years 2020-2021) confirms that the expenditure and consumption of ibrutinib (with 12.4% and 16.5% respectively; supplied by public health facilities) were higher than obinutuzumab (with 0.9% and 2.5% respectively; used in hospitals) among the list of the first 30 orphan medicines. Specifically, ibrutinib was in the second position while obinutuzumab in the twenty-eighth one (Italian Medicine Agency AIFA, 2022).

From our analyses, ibrutinib showed a higher reporting probability of ICSRs with events belonging to the SOC “Cardiac disorders” and “Vascular disorders” compared with obinutuzumab. In particular, a grow incidence of atrial fibrillation, ventricular arrhythmias and sudden death with BTK inhibitors was identified in the literature (Lampson et al., 2017; Salem et al., 2019; Christensen et al., 2022). This might be a drug class effect linked with the inhibition of both BTK-mediated pathways and multiple off-target kinases (Palma et al., 2021). Although data support the increased CV toxicity with BTKis as a class effect, newer inhibitors appear to have lower CV risk than ibrutinib due to their higher selectivity for BTK. However, this aspect has to be deepened furtherly. To our knowledge, no other studies have previously compared the risk of CV events between ibrutinib and obinutuzumab. However, the safety profile of these drugs needs to be viewed in a larger context, in which we have two different therapeutic regimens used in clinical practice. On one hand, the continuous therapy with BTK inhibitors and, on the other hand, the time-limited therapy with obinutuzumab and venetoclax/chlorambucil. Indeed, the continuous long-term administration of BTK inhibitors can favor the onset of adverse events or drug resistance (Burger, 2019). Evidence generally supported the use of time-limited obinutuzumab and venetoclax/chlorambucil over BTK inhibitors monotherapy as first therapy, considering the similar efficacy, the lower costs, and the lower adverse events (Bennett et al., 2023). However, the easier oral administration with BTK inhibitors may impede the consideration of aforementioned aspects.

The onset of CV events may limit the duration of the optimal care leading to treatment discontinuation with the risk of worsening of CLL and life-threatening CV outcomes. However, despite over a decade of research, the biological mechanisms underlying ibrutinib cardiotoxicity remain unclear. Dong et al. hypothesized multiple mechanisms with both on- and off-target effects of ibrutinib (Dong et al., 2022).

Recent studies found plausible mechanisms that may be behind the association between ibrutinib and cardiovascular adverse events, such as hypertension and atrial fibrillation. Ibrutinib has been demonstrated to inhibit the pathway of phosphoinositide 3-kinase (PI3K)/Akt, including indirect downregulation of PI3K (p110α) (McMullen et al., 2014; Dickerson et al., 2019). PI3K (p110α) is a protein with protective effects in the onset of cardiac dysfunction. Indeed, the reduction of PI3K activity (p110α) in atrial samples of patients was related to the development of AF in humans (Pretorius et al., 2009). Moreover, the inhibition of PI3K-(p110α) was related to the development of vascular remodeling and fibrosis (Dickerson et al., 2019).

According to the literature on the first-generation BTK inhibitors, in our analysis the most reported CV events were atrial fibrillation, haemorrhage, and hypertension (44.1%) (Larsson et al., 2022; Tang et al., 2022).

Atrial fibrillation occurs in 5%–16% of patients, most commonly in those with CV risk factors (e.g., older age, male sex, history of atrial fibrillation, hyperlipidaemia, hypertension and valvular heart disease) (Brown et al., 2017; Jiang et al., 2019). Atrial fibrillation is also the most frequent cause for toxicity-related discontinuation of ibrutinib (Xiao et al., 2020). We found a higher reporting of atrial fibrillation with ibrutinib therapy. Accordingly, a retrospective study found a lower development of atrial fibrillation in patients affected by CLL treated with obinutuzumab plus chlorambucil than ibrutinib in spite of a not statistically significant difference (2% vs. 9%, *p* = 0.0813) (Visentin et al., 2022).

Ibrutinib is even associated with bleeding due to its effect on several distinct platelet signaling pathways. Significant bleeding complications may emerge with concomitant use of anticoagulants or antiplatelets. Limited data are available to help clinicians on the use of ibrutinib in patients at high risk for bleeding. Patients and HCPs should be warned of not using nonsteroidal anti-inflammatory drugs, fish oils, vitamin E, vitamin K antagonists, and aspirin-containing products, and consider the substitution of ibrutinib with another drug if dual antiplatelet therapy is needed (Shatzel et al., 2017).

Hypertension is another of the most common CV event with ibrutinib, reported in up to 30% of patients in clinical trials and up to 80% of patients in real-world studies (Christensen et al., 2022).

On the contrary, hypotension is most reported with obinutuzumab according to the literature and the Summary of Product Characteristic (SmPC) data that include it among signs of infusion reaction (Evans and Clemmons, 2015; European Medicines Agency (EMA), 2019).

Moreover, a current disproportionality analysis from Food and Drug Administration Adverse Event Reporting System (FAERS) database identified positive signals for 10 CV events related to ibrutinib (supraventricular tachyarrhythmias, haemorrhagic central nervous system vascular conditions, ventricular tachyarrhythmias, cardiac failure, ischaemic central nervous system vascular conditions, cardiomyopathy, conduction defects, myocardial infarction, myocardial infarction disorders of sinus node function, and torsade de pointes/QT prolongation). In this analysis, Zheng et al. reported that the signal for disorders of sinus node function was observed for the first time and it could be a new adverse effect of ibrutinib. Our findings showed only one case associated with the combination ibrutinib/Obinutuzumab (Zheng et al., 2023).

In our analysis, the risk of CV events related to ibrutinib was consistent with that reported in previous studies, with no new CV safety concerns identified. However, we believe that our results can provide important information on the significant risk of CV events related to ibrutinib among real-world CLL patients, outside the setting of clinical trials. In the era of second-generation BTK inhibitors, which seem to induce less frequently cardiotoxicity than the first generation (St-Pierre and Ma, 2022), our findings suggest the need to move towards a better management of ibrutinib-related CV events in the setting of therapy optimization, especially for elderly and frail patients affected by CLL.

## 5 Strengths and limits

The main strength of our study is the use of the EV database that allows to detect a wide range of safety cases in treatment with ibrutinib or/and obinutuzumab for a rare disease such as CLL. Moreover, the use of pharmacocovigilance data is of free access and helpful to better characterize the drug safety profile.

Several limits need to be identified for the EV analysis. Some suspected CV cases may not be reported to the national drug authorities, and therefore not submitted to EV (underreporting).

Another limitation is the possibility of a non-homogeneous and incomplete information reported in ICSRs (lack of information on age, drug dosing, comorbid conditions, and concomitant drugs), without also the possibility to check for clinical or laboratory tests that can help to justify the reported diagnosis. A relevant limit of the study is the lack of a clinical context around the events reported. In fact, considering our data source, the analyses could not include initial patient comorbidities or CLL clinicopathological characteristics. Moreover, we cannot retrieve information on the exact dates of administration and development of the event. Therefore, no time to event was computable. Finally, the exact denominator of patients exposed to ibrutinib or obinutuzumab cannot be considered in EV. Instead, the total number of events for each drug can be used as a denominator for disproportionality analyses in pharmacovigilance databases. Although the two drugs have been centrally authorized by EMA in the same year (2014), in absence of consumption and prescription data, we cannot exclude that differences in the number of ICSRs and in the RORs were due to differences in their consume.

## 6 Conclusion

Although the limitations of spontaneous reporting system (e.g., underreporting, reporting bias) should be considered, our experience regarding the analysis of ICSRs reported in EV has allowed us to confirm, according to what reported in literature, a higher ROR of CV events, especially atrial fibrillation, haemorrhage, and hypertension, in patients exposed to ibrutinib in comparison to another pharmacological treatment for CLL (Obinutuzumab).

Our results have demonstrated that 24% and 7% of all ICSRs reporting ibrutinib or obinutuzumab described the occurrence of CV events. Moreover, most reported CV events were serious and were associated with male sex. Indeed, such CV adverse events may have a strong impact on the quality of life of oncological patients, taking account that the majority is also elderly. Thus, we believe that the pharmacovigilance activities can play a key role in the re-definition of the risk/benefit profile of ibrutinib (and/or of its class) over the time, if the CV toxicity should significantly increase.

Moreover, further research is needed to understand, explain and manage correctly CV events related to ibrutinib within an overall healthcare management of patients affected by CLL. This approach is important to avoid the early discontinuation and the preclusion of a revolutionary treatment for both naïve or relapsed/refractory CLL patients.

## Data Availability

The datasets presented in this study can be found in online repositories. The names of the repository/repositories and accession number(s) can be found below: https://www.adrreports.eu/it/search.html.
