# Supplementary material for: Which is the top player for the cardiovascular safety? ibrutinib vs. obinutuzumab in CLL
Source: Front Pharmacol. 2023 Aug 16;14:1229304. doi: 10.3389/fphar.2023.1229304 (PMC10467285; doi:10.3389/fphar.2023.1229304)
Supplement: Supplementary file 1 [file DataSheet1.docx]

Supplementary Material

Which is the top player for the cardiovascular safety?

Ibrutinib vs. Obinutuzumab in CLL

**Annamaria Mascolo and Raffaella Di Napoli* and Nunzia Balzano, Elena D’Alessio, Imma Izzo, Francesco Rossi, Giuseppe Paolisso, Annalisa Capuano and Liberata Sportiello.**

*** Correspondence:**^*^ Raffaella Di Napoli - Campania Regional Centre for Pharmacovigilance and Pharmacoepidemiology - Department of Experimental Medicine – Section of Pharmacology “L. Donatelli”, University of Campania “Luigi Vanvitelli”, Via Costantinopoli 16 – 80138 Naples (Italy); Phone: 00390815665805; Email: [raffaella.dinapoli@unicampania.it](mailto:raffaella.dinapoli@unicampania.it)

**Supplementary Table 1:** Other cardiovascular events listed in Individual Case Safety Reports (ICSRs) with ibrutinib and/or obinutuzumab as suspected drug and reported in Eudravigilance from January 1st. 2014 to September 30th. 2022.

| **Cardiovascular events** | **Ibrutinib** | **Ibrutinib/Obinituzumab** | **Obinituzumab** | **Total** |
| --- | --- | --- | --- | --- |
| Atrial fibrillation | 885 | 4 | 24 | 913 |
| Haemorrhage | 197 | 0 | 4 | 201 |
| Hypertension | 140 | 1 | 32 | 173 |
| Hypotension | 51 | 2 | 107 | 160 |
| Haematoma | 115 | 0 | 1 | 116 |
| Cardiac failure | 80 | 1 | 8 | 89 |
| Cardiac failure congestive | 82 | 0 | 5 | 87 |
| Myocardial infarction | 52 | 0 | 18 | 70 |
| Tachycardia | 28 | 1 | 33 | 62 |
| Arrhythmia | 54 | 0 | 2 | 56 |
| Cardiac disorder | 41 | 1 | 11 | 53 |
| Palpitations | 45 | 1 | 6 | 52 |
| Cardiac arrest | 38 | 0 | 11 | 49 |
| Atrial flutter | 44 | 0 | 0 | 44 |
| Pericardial effusion | 39 | 0 | 1 | 40 |
| Flushing | 7 | 0 | 23 | 30 |
| Acute myocardial infarction | 17 | 0 | 8 | 25 |
| Thrombosis | 23 | 1 | 1 | 25 |
| Ventricular fibrillation | 24 | 0 | 1 | 25 |
| Ventricular tachycardia | 25 | 0 | 0 | 25 |
| Pericarditis | 24 | 0 | 0 | 24 |
| Cardiomyopathy | 21 | 0 | 1 | 22 |
| Deep vein thrombosis | 21 | 1 | 0 | 22 |
| Bradycardia | 11 | 0 | 10 | 21 |
| Cardio-respiratory arrest | 14 | 0 | 5 | 19 |
| Pericardial haemorrhage | 19 | 0 | 0 | 19 |
| Ventricular extrasystoles | 16 | 0 | 2 | 18 |
| Lymphoedema | 14 | 0 | 1 | 15 |
| Angina pectoris | 6 | 0 | 8 | 14 |
| Cardiac tamponade | 13 | 0 | 0 | 13 |
| Hot flush | 7 | 0 | 6 | 13 |
| Supraventricular tachycardia | 11 | 0 | 2 | 13 |
| Hypertensive crisis | 11 | 0 | 1 | 12 |
| Coronary artery disease | 7 | 0 | 4 | 11 |
| Cardiac flutter | 10 | 0 | 0 | 10 |
| Internal haemorrhage | 9 | 0 | 0 | 9 |
| Acute coronary syndrome | 6 | 0 | 2 | 8 |
| Cardiogenic shock | 8 | 0 | 0 | 8 |
| Circulatory collapse | 4 | 0 | 4 | 8 |
| Orthostatic hypotension | 6 | 0 | 2 | 8 |
| Sinus tachycardia | 7 | 0 | 1 | 8 |
| Supraventricular extrasystoles | 8 | 0 | 0 | 8 |
| Vasculitis | 8 | 0 | 0 | 8 |
| Atrioventricular block | 7 | 0 | 0 | 7 |
| Blood pressure fluctuation | 5 | 0 | 2 | 7 |
| Peripheral coldness | 7 | 0 | 0 | 7 |
| Cardiomegaly | 6 | 0 | 0 | 6 |
| Congestive cardiomyopathy | 6 | 0 | 0 | 6 |
| Haemodynamic instability | 5 | 0 | 1 | 6 |
| Infarction | 4 | 0 | 2 | 6 |
| Left ventricular dysfunction | 6 | 0 | 0 | 6 |
| Ventricular arrhythmia | 6 | 0 | 0 | 6 |
| Aortic stenosis | 5 | 0 | 0 | 5 |
| Arrhythmia supraventricular | 4 | 0 | 1 | 5 |
| Bundle branch block right | 5 | 0 | 0 | 5 |
| Cardiac fibrillation | 4 | 0 | 1 | 5 |
| Cardiovascular disorder | 4 | 0 | 1 | 5 |
| Coronary artery occlusion | 4 | 1 | 0 | 5 |
| Cyanosis | 1 | 0 | 4 | 5 |
| Extrasystoles | 5 | 0 | 0 | 5 |
| Ischaemic cardiomyopathy | 5 | 0 | 0 | 5 |
| Right ventricular failure | 5 | 0 | 0 | 5 |
| Shock | 3 | 0 | 2 | 5 |
| Sinus bradycardia | 4 | 0 | 1 | 5 |
| Stress cardiomyopathy | 5 | 0 | 0 | 5 |
| Aortic aneurysm | 4 | 0 | 0 | 4 |
| Aortic valve stenosis | 3 | 0 | 1 | 4 |
| Atrioventricular block first degree | 3 | 1 | 0 | 4 |
| Atrioventricular block second degree | 4 | 0 | 0 | 4 |
| Cardiopulmonary failure | 3 | 0 | 1 | 4 |
| Hyperaemia | 0 | 0 | 4 | 4 |
| Left ventricular hypertrophy | 4 | 0 | 0 | 4 |
| Mitral valve incompetence | 4 | 0 | 0 | 4 |
| Myocardial ischaemia | 3 | 0 | 1 | 4 |
| Pallor | 2 | 0 | 2 | 4 |
| Torsade de pointes | 4 | 0 | 0 | 4 |
| Aortic aneurysm rupture | 3 | 0 | 0 | 3 |
| Aortic valve disease | 3 | 0 | 0 | 3 |
| Atrioventricular block complete | 3 | 0 | 0 | 3 |
| Bundle branch block left | 3 | 0 | 0 | 3 |
| Cardiac failure chronic | 3 | 0 | 0 | 3 |
| Conduction disorder | 3 | 0 | 0 | 3 |
| Embolism | 2 | 0 | 1 | 3 |
| Left ventricular failure | 3 | 0 | 0 | 3 |
| Myocarditis | 3 | 0 | 0 | 3 |
| Peripheral arterial occlusive disease | 3 | 0 | 0 | 3 |
| Peripheral venous disease | 3 | 0 | 0 | 3 |
| Poor peripheral circulation | 2 | 0 | 1 | 3 |
| Superficial vein thrombosis | 3 | 0 | 0 | 3 |
| Tachyarrhythmia | 3 | 0 | 0 | 3 |
| Angina unstable | 2 | 0 | 0 | 2 |
| Angiopathy | 2 | 0 | 0 | 2 |
| Aortic dissection | 2 | 0 | 0 | 2 |
| Aortic valve incompetence | 1 | 0 | 1 | 2 |
| Arteriospasm coronary | 1 | 0 | 1 | 2 |
| Bifascicular block | 2 | 0 | 0 | 2 |
| Blue toe syndrome | 2 | 0 | 0 | 2 |
| Bradyarrhythmia | 1 | 0 | 1 | 2 |
| Capillary fragility | 2 | 0 | 0 | 2 |
| Cardiac failure acute | 2 | 0 | 0 | 2 |
| Cardiac valve disease | 2 | 0 | 0 | 2 |
| Cardiotoxicity | 2 | 0 | 0 | 2 |
| Coronary artery dissection | 2 | 0 | 0 | 2 |
| Essential hypertension | 2 | 0 | 0 | 2 |
| Heart valve incompetence | 1 | 0 | 1 | 2 |
| Hypertensive urgency | 1 | 1 | 0 | 2 |
| Ischaemia | 2 | 0 | 0 | 2 |
| Labile blood pressure | 1 | 0 | 1 | 2 |
| Mitral valve prolapse | 2 | 0 | 0 | 2 |
| Phlebitis | 1 | 0 | 1 | 2 |
| Shock haemorrhagic | 2 | 0 | 0 | 2 |
| Ventricular hypokinesia | 1 | 1 | 0 | 2 |
| Accelerated hypertension | 1 | 0 | 0 | 1 |
| Aneurysm | 1 | 0 | 0 | 1 |
| Aortic arteriosclerosis | 0 | 0 | 1 | 1 |
| Aortic disorder | 0 | 0 | 1 | 1 |
| Aortic intramural haematoma | 1 | 0 | 0 | 1 |
| Aortic thrombosis | 1 | 0 | 0 | 1 |
| Arrhythmic storm | 1 | 0 | 0 | 1 |
| Arterial occlusive disease | 1 | 0 | 0 | 1 |
| Arteriosclerosis | 0 | 0 | 1 | 1 |
| Arteriosclerosis coronary artery | 1 | 0 | 0 | 1 |
| Artery dissection | 1 | 0 | 0 | 1 |
| Behcet's syndrome | 1 | 0 | 0 | 1 |
| Bleeding varicose vein | 1 | 0 | 0 | 1 |
| Cardiac dysfunction | 1 | 0 | 0 | 1 |
| Cardiomyopathy acute | 1 | 0 | 0 | 1 |
| Carditis | 1 | 0 | 0 | 1 |
| Coronary artery insufficiency | 0 | 0 | 1 | 1 |
| Coronary ostial stenosis | 1 | 0 | 0 | 1 |
| Cryoglobulinaemia | 1 | 0 | 0 | 1 |
| Diastolic dysfunction | 1 | 0 | 0 | 1 |
| Dry gangrene | 1 | 0 | 0 | 1 |
| Embolism venous | 1 | 0 | 0 | 1 |
| Endothelial dysfunction | 1 | 0 | 0 | 1 |
| Eosinophilic myocarditis | 1 | 0 | 0 | 1 |
| Extremity necrosis | 1 | 0 | 0 | 1 |
| Haemorrhagic vasculitis | 1 | 0 | 0 | 1 |
| Hypovolaemic shock | 1 | 0 | 0 | 1 |
| Intermittent claudication | 1 | 0 | 0 | 1 |
| Jugular vein thrombosis | 1 | 0 | 0 | 1 |
| Malignant hypertension | 1 | 0 | 0 | 1 |
| May-Thurner syndrome | 1 | 0 | 0 | 1 |
| Mitral valve stenosis | 1 | 0 | 0 | 1 |
| Myocardial injury | 0 | 0 | 1 | 1 |
| Myocardial necrosis | 1 | 0 | 0 | 1 |
| Myopericarditis | 1 | 0 | 0 | 1 |
| Orthostatic hypertension | 1 | 0 | 0 | 1 |
| Pericarditis constrictive | 1 | 0 | 0 | 1 |
| Peripheral vascular disorder | 1 | 0 | 0 | 1 |
| Phlebitis superficial | 1 | 0 | 0 | 1 |
| Pleuropericarditis | 1 | 0 | 0 | 1 |
| Polyarteritis nodosa | 1 | 0 | 0 | 1 |
| Raynaud's phenomenon | 1 | 0 | 0 | 1 |
| Right ventricular dysfunction | 1 | 0 | 0 | 1 |
| Sinoatrial block | 1 | 0 | 0 | 1 |
| Sinus arrest | 0 | 1 | 0 | 1 |
| Sinus arrhythmia | 1 | 0 | 0 | 1 |
| Sinus node dysfunction | 0 | 1 | 0 | 1 |
| Superior vena cava syndrome | 0 | 0 | 1 | 1 |
| Systolic dysfunction | 1 | 0 | 0 | 1 |
| Thrombophlebitis | 1 | 0 | 0 | 1 |
| Trifascicular block | 1 | 0 | 0 | 1 |
| Varicose vein | 1 | 0 | 0 | 1 |
| Vasodilatation | 1 | 0 | 0 | 1 |
| Venous occlusion | 1 | 0 | 0 | 1 |
| Venous thrombosis | 1 | 0 | 0 | 1 |
| Venous thrombosis limb | 1 | 0 | 0 | 1 |
| Ventricular dysfunction | 1 | 0 | 0 | 1 |
| Ventricular flutter | 1 | 0 | 0 | 1 |
| Total | 2,514 | 19 | 383 | 2,916 |

**Supplementary Figure 1:** the annual spontaneous reporting trend of ibrutinib CV ICSRs and the annual percentage change reported in Eudravigilance from January 1st. 2014 to September 30th. 2022


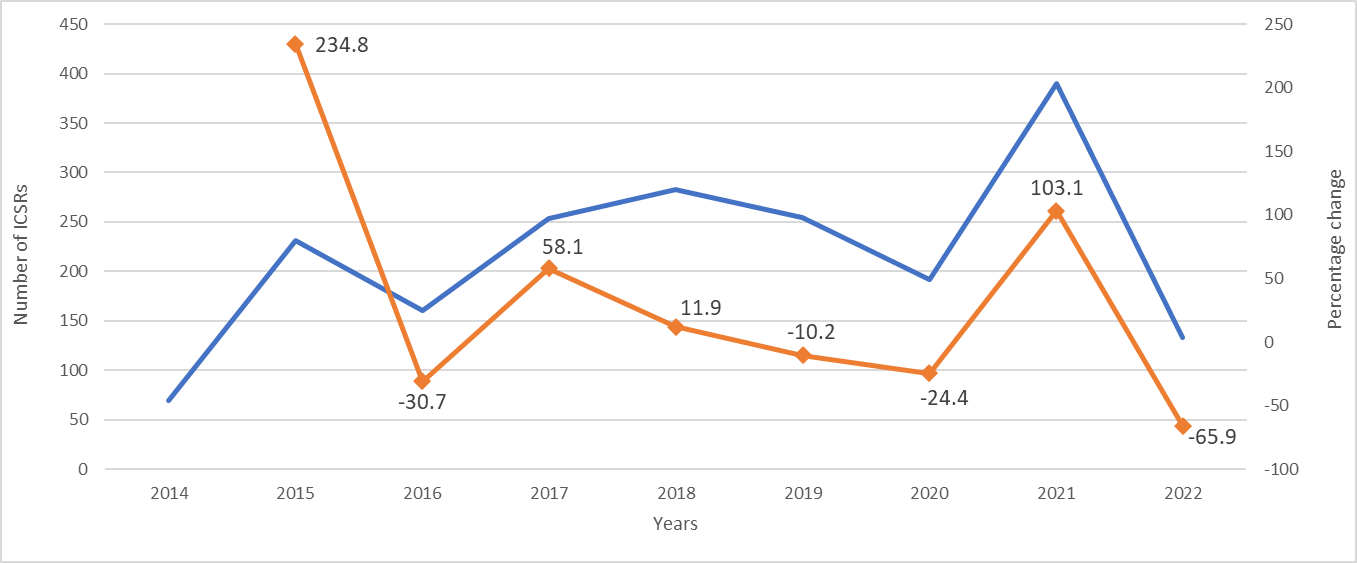


**Supplementary Figure 2:** the annual spontaneous reporting trend of obinutuzumab CV ICSRs and the annual percentage change reported in Eudravigilance from January 1st. 2014 to September 30th. 2022


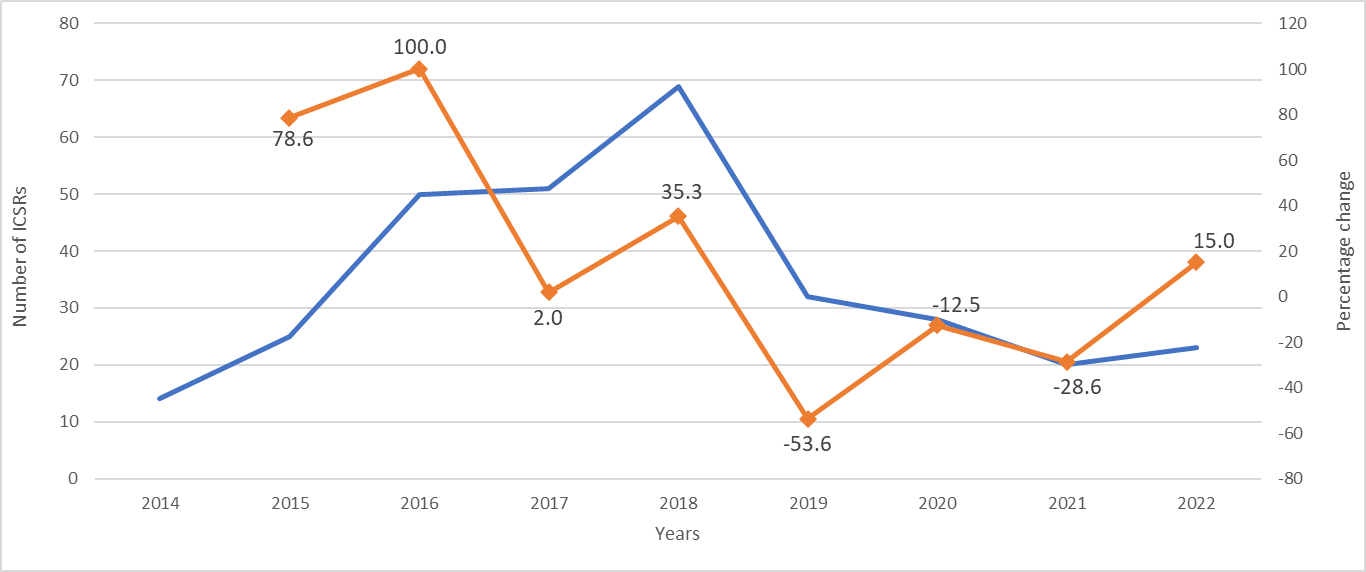


**Supplementary Figure 3:** the annual spontaneous reporting trend of fatal outcomes and the annual percentage change reported in Eudravigilance from January 1st. 2014 to September 30th. 2022


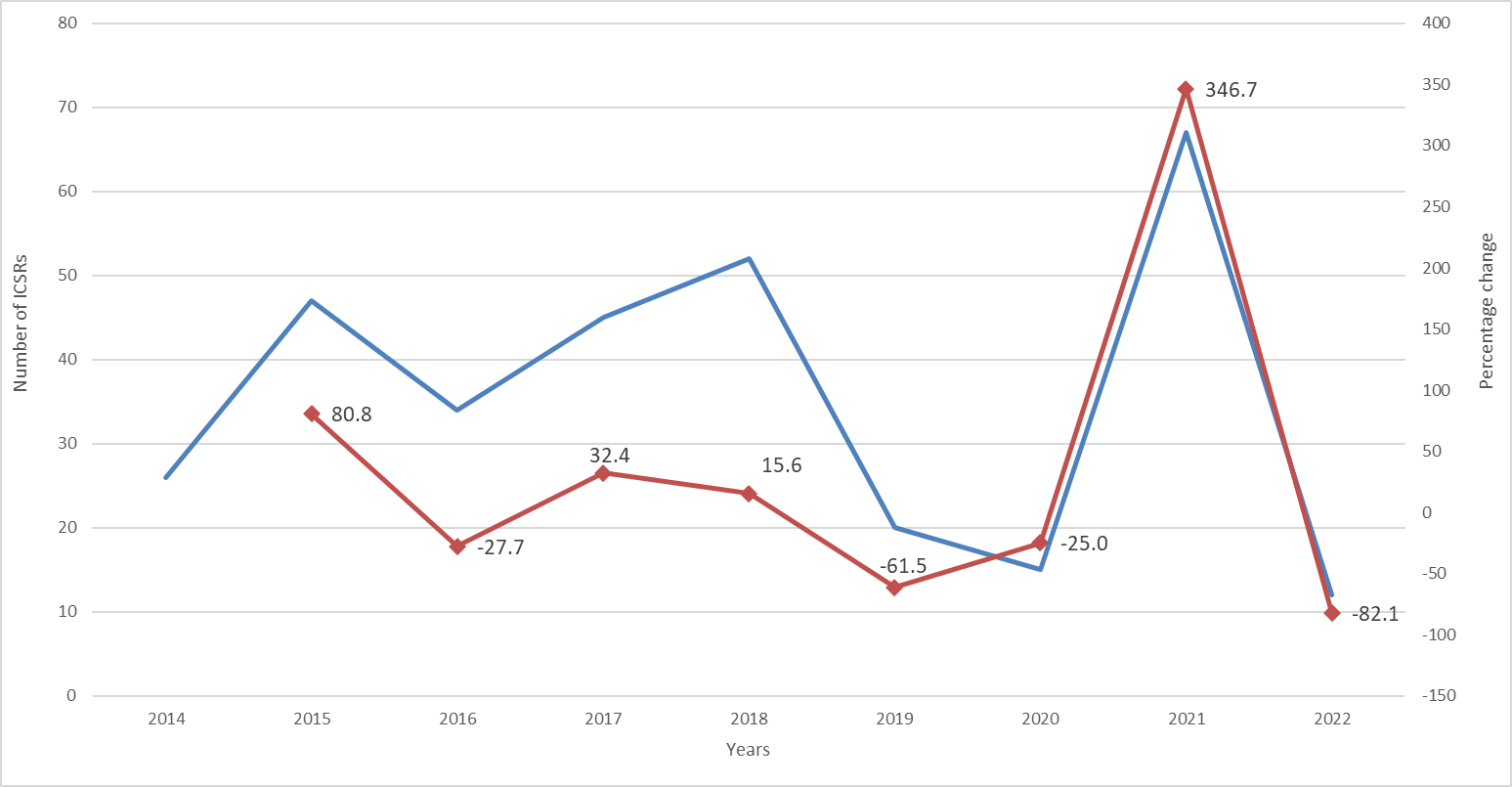


**
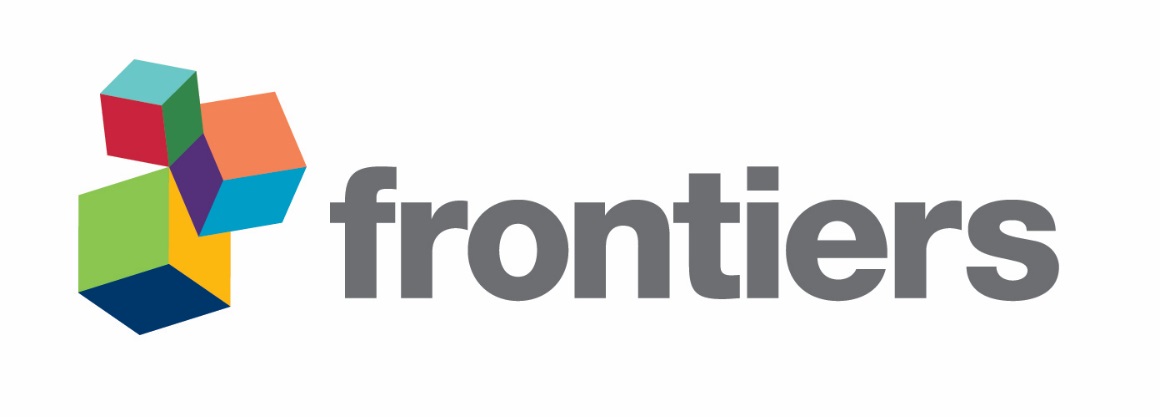
**
